# Mechanisms of bacterial and fungal community assembly in leaf miners during transition from natural to laboratory environments

**DOI:** 10.3389/fmicb.2024.1424568

**Published:** 2024-07-18

**Authors:** Yu-Xi Zhu, Xin-Yu Wang, Tian-Yue Yang, Huan-Huan Zhang, Tong-Pu Li, Yu-Zhou Du

**Affiliations:** ^1^Department of Entomology, College of Plant Protection, Yangzhou University, Yangzhou, China; ^2^Institute of Vegetable, Tibet Academy of Agriculture and Animal Husbandry Science, Lhasa, China; ^3^Co-Innovation Center for Sustainable Forestry in Southern China, College of Forestry, Nanjing Forestry University, Nanjing, Jiangsu, China

**Keywords:** *Liriomyza huidobrensis*, community assembly, co-occurrence networks, null model, neutral model

## Abstract

Environmental heterogeneity partly drives microbial succession in arthropods, while the microbial assembly mechanisms during environmental changes remain largely unknown. Here, we explored the temporal dynamics and assembly mechanisms within both bacterial and fungal communities in *Liriomyza huidobrensis* (Blanchard) during the transition from field to laboratory conditions. We observed a decrease in bacterial diversity and complexity of bacterial-fungal co-occurrence networks in leaf miners transitioning from wild to captive environments. Both neutral and null models revealed that stochastic processes, particularly drift (contributing over 70%), play a crucial role in governing bacterial and fungal community assembly. The relative contribution of ecological processes such as dispersal, drift, and selection varied among leaf miners transitioning from wild to captive states. Furthermore, we propose a hypothetical scenario for the assembly and succession of microbial communities in the leaf miner during the short- and long-term transition from the wild to captivity. Our findings suggest that environmental heterogeneity determines the ecological processes governing bacterial and fungal community assembly in leaf miners, offering new insights into microbiome and mycobiome assembly mechanisms in invasive pests amidst environmental change.

## 1 Introduction

Insect colonies are commonly established and maintained under laboratory conditions for various purposes, including research, edible products, or pest management programs (Gong et al., 2020; Hawkey et al., 2021; Nikolouli et al., 2022). An increasing number of studies on the microbiota of different insect systems have demonstrated that long-term adaptation to the laboratory environment significantly alters the host microbiome and mycobiome, with consequential effects on host fitness or reproduction (Hegde et al., 2018; Tinker and Ottesen, 2021; Majumder et al., 2022; Nikolouli et al., 2022). Understanding the dynamics and succession of microbiome and mycobiome assembly during the transition of insects from the wild to the laboratory is crucial for the success of their applications or for comprehending host-microbiota evolution in response to environmental changes. However, the mechanisms underlying their assembly remain largely unknown and pose a major challenge in insect microbiology.

Selection, dispersal and drift have been proposed to be the primary processes governing microbial community assembly (Zhou and Ning, 2017; Coyte et al., 2021). Among these processes, selection is considered purely deterministic, leading to community convergence or divergence depending on environmental conditions, while drift results in stochastic community dispersion. Dispersal is thought to encompass both deterministic and stochastic elements, depending on the magnitude: homogeneous dispersal and dispersal limitation lead to community convergence and divergence, respectively. For many insects, both deterministic and stochastic forces collectively drive host bacterial or fungal community assembly (Adair et al., 2018; Ge et al., 2021; Yang et al., 2022; Zhu et al., 2022a,b). The relative importance of these processes in governing host-microbiomes remains highly debated; some studies suggest that deterministic processes, particularly selection, are crucial (Yang et al., 2022; Zhu et al., 2022b), while comparative research emphasizes stochasticity (Zhu et al., 2023). These discrepancies often arise due to differences in environment or host genetics, both of which can impact the relative contribution of each ecological process (Ge et al., 2021; Jones et al., 2022). Invasive pests often undergo significant changes in environmental conditions, potentially resulting in microbiome succession.

The invasive leaf miner *Liriomyza huidobrensis* has rapidly spread worldwide, causing significant economic losses (Weintraub et al., 2017; Zhu et al., 2022a). Insect-associated microbes may play a crucial role in host invasion and adaption in various ways (Liu et al., 2022), and leaf miner-associated microbiota is no exception. However, experimental verification through the establishment of laboratory populations is necessary. Although our previous work suggested that environmental factors influence their microbiota community (Zhu et al., 2022a), the microbiome and mycobiome assembly during the transition of hosts from the wild to the laboratory with significant environmental changes remains underappreciated, limiting our understanding of host-microbiome evolution.

To address these questions, we conducted deep sequencing of the bacterial 16S rRNA gene and the fungal ITS region of 83 samples from three wild leaf miner populations transferred from the wild to the laboratory. Initially, we explored the diversity, composition, and network structure of the bacterial and fungal community in leaf miners during environmental changes. Subsequently, we investigated the underlying mechanisms of bacterial and fungal community assembly using neutral and null model analysis and assessed the impact of variables (e.g., original host plant, location and life stage) on the host microbiome assembly process.

## 2 Materials and methods

### 2.1 Leaf miner populations and rearing

Three wild populations of the leaf miner *Liriomyza huidobrensis* were collected between March and April 2023 from three different host plant species (i.e., bean, luffa, and tomato) in two geographic locations in China (Yunnan and Xinjiang). *Liriomyza huidobrensis* pupae were placed in sterile plastic tubes. After mating, the newly emerged adults from each wild population were randomly divided into two groups: one group (Day 0, *n* = 6) constituted the wild population and was gently collected for DNA extraction, while the other adults were kept in cages with bean plants at 25°C under a long-day regime (16 h light/8 h dark). Each population of leaf miners was maintained separately. After 48 h of oviposition, the adults were removed from the cages. Subsequently, we collected eggs (3 days old), larvae (2nd instar), pupae (2 days old), and adults (0 days old) in 1.5 mL collection tubes filled with 75% (v/v) ethanol. A total of 30 eggs were pooled to form one sample, with three biological replicates per sample. For larvae, pupae, and adults, each individual was considered one replicate, with six replicates for each treatment. Each sample was surface washed with 75% ethanol and sterile water before DNA extraction.

### 2.2 DNA extractions, library preparation, and sequencing

The total DNA of insect samples was extracted using a DNeasy blood and tissue kit (Qiagen, Hilden, Germany) following the handbook. We amplified the bacterial 16S rRNA V3-V4 region and the fungal internal transcribed spacer (ITS) ITS1/2 region using the primers 341F (5′-CCTAYGGGRBGCASCAG-3′) and 806R (5′-GGACTACNNGGGTATCTAAT-3′), and ITS1F (5′-CTTGGTCATTTAGAGG AAGTAA-3′) and ITS2R (5′-GCTGCGTTCTTCATC GATGC-3′), respectively (Bing et al., 2020). PCR amplification was performed using ABI GeneAmp® 9700. For both the 16S rRNA and the ITS1/2 amplicons, three replicates for each sample were amplified using the following protocol: 95°C for 5 min, 30 cycles at 95°C for 30 s, 52°C for 30 s, 72°C for 45 s, and a final extension at 72°C for 10 min. The purified PCR products were normalized to equimolar amounts before sequencing. The 16S rRNA and the ITS1/2 amplicon library were sequenced separately using 250-bp paired-end reads on an Illumina MiSeq 2500 platform by Shanghai Biozeron Co., Ltd. The raw sequence data were filtered for quality control using the DADA2 pipeline. After quality filtering and chimera removal, we obtained 6,507,086 clear reads of bacterial amplicon sequences and 7,426,321 clear reads of fungal amplicon sequences. Bacterial and fungal amplicon sequence variants (ASVs) were classified against the SILVA database and Unite database. For diversity analysis, all samples were rarefied to the same normalized sequencing depth.

### 2.3 Microbial diversity analysis

The Shannon alpha diversity index was calculated using the vegan package in R version 3.6.2. To assess significant differences in fungal or bacterial diversity among leaf miner stages, a nonparametric statistical test was conducted. Permutational multivariate ANOVA (PERMANOVA) was performed to evaluate the disparity in community composition among leaf miner stages using the adonis function of the vegan package, based on Bray–Curtis distances. Redundancy analysis was employed to examine the relative contribution of host-related factors to the overall compositional variation in the bacterial or fungal community (Van Den Wollenberg, 1977), and the results were visualized using the ggplot2 package.

### 2.4 Co-occurrence networks analysis

Bacterial and fungal co-occurrence networks were constructed to assess the coexistence of bacteria, fungi, or bacterial-fungal interactions across various groups. All network analyses were conducted using the Spie-cEasi package, and the results were visualized using the ggClusterNet package. Only robust (*ρ* > 0.8 or *ρ* < −0.8) and statistically significant (*p* < 0.05) correlations were retained for visualization. The vegan and igraph packages were used to evaluate various network parameters (Zhu et al., 2022c).

### 2.5 Microbial assembly analysis

We utilized parallel null and neutral model analyses to investigate the mechanisms driving microbial community assembly (Sloan et al., 2006; Zhou and Ning, 2017; Zhu et al., 2022c). Firstly, we employed the Sloan neutral model to estimate the occurrence frequency of each bacterial or fungal ASV in the metacommunity, using the stats and minpack.lm packages. The calculation of 95% confidence intervals for the Sloan model helped detect whether the frequency of ASVs deviated from the expected data distribution, with the parameter *R*^2^ indicating the goodness of fit of the model.

Secondly, we evaluated the contribution of various deterministic and stochastic processes to insect community assembly through null model analysis. We constructed bacterial and fungal phylogenetic trees using Ghost Tree (Fouquier et al., 2016) and FastTree2 (Price et al., 2010), respectively. Subsequently, we calculated the beta Nearest Taxon Index (βNTI) by comparing the observed β-mean nearest taxon distance (βMNTD) to the mean of a null distribution of βMNTD (999 randomizations). |βNTI| ≥ 2 denotes deterministic processes dominating in shaping communities, while |βNTI| < 2 indicates a stronger influence from stochastic processes. We then combined βNTI with Bray-Curtis-based Raup–Crick indices (RCI) to estimate the relative contribution of ecological processes in community assembly. βNTI < −2 and βNTI >2 indicate homogeneous selection and variable selection, respectively; |βNTI| < 2 with RCI < −0.95 or > 0.95 suggests that the deviation was contributed by homogenizing dispersal or dispersal limitation; |RCI| < 0.95 and |βNTI| < 2 indicate drift in driving the composition of the microbiota.

### 2.6 Variables and microbial assembly

To test the major factors, including locations, host plants, and developmental stage, that affected bacterial and fungal assembly processes, a Mantel test was carried out to investigate the relationship between βNTI values and these variables using the vegan package in R (Zhu et al., 2022b).

## 3 Results

### 3.1 Changes in bacterial and fungal community diversity and structure under laboratory conditions

**Figure 1:**
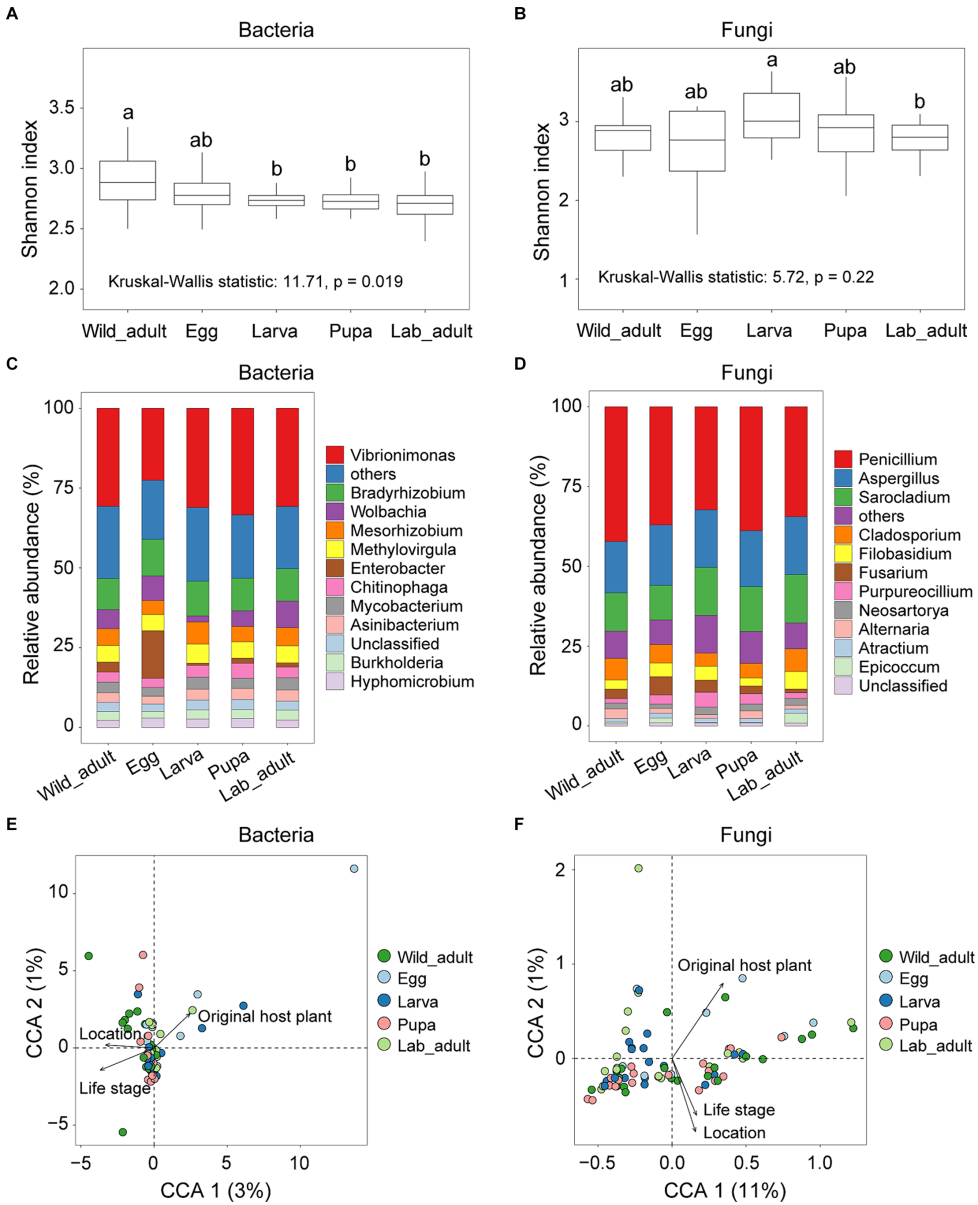
Diversity and composition of bacterial and fungal communities in leaf miners transferred from the wild to the laboratory. **(A)** Shannon index of bacterial diversity and **(B)** fungal diversity in each group. Letters above the whiskers denote significant differences between each group based on pairwise comparison. **(C)** Relative abundances of bacterial genera and **(D)** fungal genera in microbial composition among each groups. The relative contribution of host-related factors, including original host plant, life stage, and location to the overall compositional variation in the bacterial **(E)** or fungal community **(F)**.

The Shannon diversity index of bacterial communities exhibited significant differences across wild adults and various developmental stages (egg, larva, pupa, and adult) in the laboratory (Kruskal-Wallis statistic: 11.71, *p* = 0.019; Figure 1A), whereas no significant differences were found for fungal communities (Kruskal-Wallis statistic: 5.72, *p* = 0.22; Figure 1B). The diversity of the bacterial community tended to decrease from the field to the laboratory (Figure 1A). There was no significant correlation between the Shannon index of bacteria and fungi in all samples (*r*^2^ = 0.042, *p* = 0.062; Supplementary Figure S1).

Bacterial community composition, rather than fungal community composition, varied significantly among life stages (ANOSIM, Bacteria: *r* = 0.058, *p* = 0.019; Fungi, *r* = 0.021, *p* = 0.17). The relative abundance of the dominant bacterial genera (e.g., *Vibrionimonas* and *Wolbachia*) and fungal genera (e.g., *Penicillium* and *Sarocladium*) differed among all life stages (*p* < 0.001 for all cases; Figures 1C,D). Redundancy analysis indicated that just 4.21 and 20.84% variance in bacterial and fungal communities, respectively, could be explained by all examined factors (Figures 1E,F).

### 3.2 Bacterial and fungal networks in leaf miners transferring from the field to the laboratory

**Figure 2:**
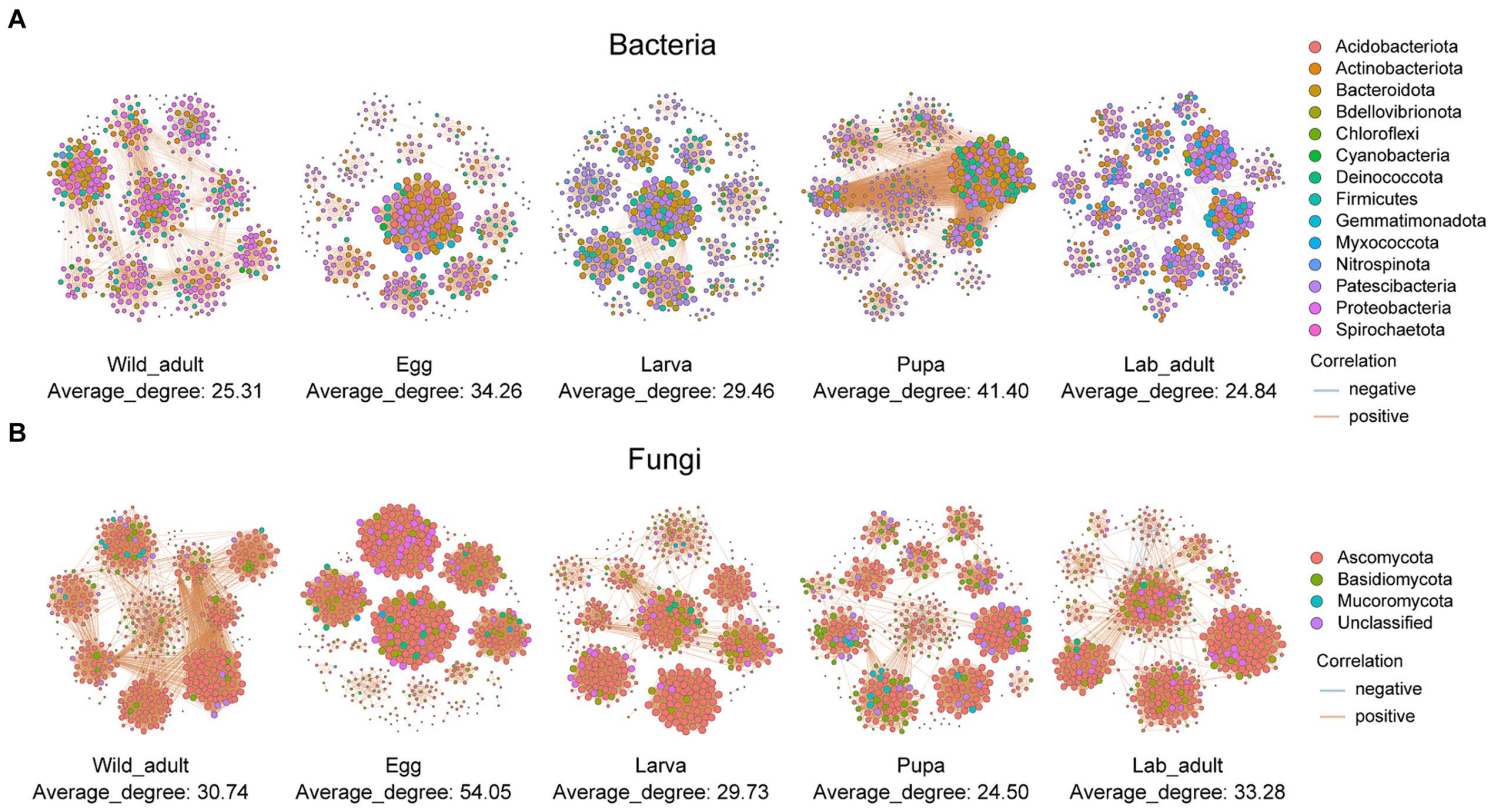
Co-occurrence networks of bacteria **(A)** and fungi **(B)** in leaf miners transferred from the wild to the laboratory. Blue and red lines indicate significant negative and positive correlations. The sizes of the points represent the relative abundances of ASVs in each microbial community.

When integrating three populations, we observed a fluctuation trend in the network complexity of both bacterial and fungal communities, as indicated by the average degree and the number of connections, from wild adults to different developmental stages (i.e., egg, larva, pupa, and adult) in the laboratory (Figure 2; Supplementary Table S1). There were slight differences in the complexity of bacterial and fungal networks between wild adults and lab adults (bacteria: average degree—25.31 vs. 24.85, number of edges—6,023 vs. 6,063; fungi: average degree—30.74 vs. 33.28, number of edges—7,408 vs. 8,034) (Figure 2; Supplementary Table S1). Positive correlations dominated in both bacterial and fungal networks from leaf miners at each life stage. The bacterial network exhibited greater complexity than the fungal network in the same host category, except for the pupa stage (Figure 2; Supplementary Table S1).

The complexity of networks of bacteria-fungi in each host tended to decrease from the wild adult to developmental stages in the laboratory (Supplementary Figure S2; Supplementary Table S2). The average degree and connections number in bacteria-fungi co-occurrence networks were noticeably higher in wild adults (average degree: 7.80, *n* = 1,615) than in lab adults (average degree: 5.99, *n* = 1,201). These results imply that rapid environmental shifts may lead to a reduction in the network complexity.

### 3.3 Bacterial and fungal assembly processes in leaf miners transferring from the field to the laboratory

**Figure 3:**
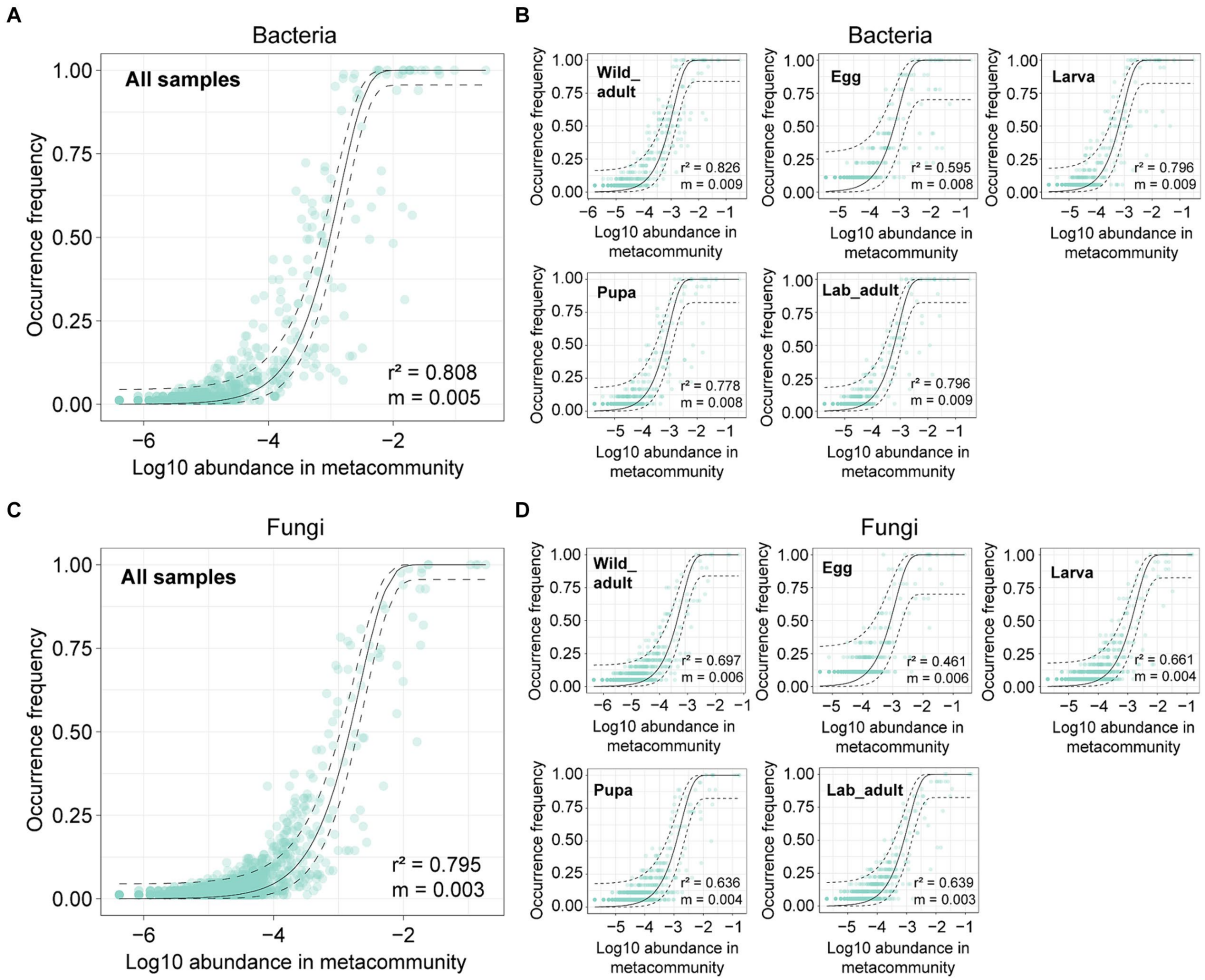
Fit of a neutral model on bacterial and fungal community assembly in all leaf miner samples **(A,C)** or each life stage **(B,D)**. The solid and dashed lines represent the predicted occurrence frequency and 95% confidence interval of the neutral model, respectively. *m* denotes the estimated migration rate, and *r*^2^ denotes the fit to the neutral model.

We first employed the neutral model to investigate the neutral processes in governing leaf miner bacterial and fungal communities. For all samples, the occurrence frequency of both bacterial and fungal ASVs within leaf miners fit the neutral model (bacteria: *r*^2^ = 0.808; fungi: *r*^2^ = 0.795) (Figures 3A,C). The majority of bacterial or fungal ASVs fell within the 95% confidence interval for the neutral prediction range. When analyzed separately for each leaf stage, the frequency of both bacterial and fungal ASVs strongly adhered to the neutral model, with goodness of fit values ranging from 0.461 to 0.826 (Figures 3B,D). These results suggest that stochastic processes play a more significant role than deterministic forces in determining leaf miner bacterial and fungal community assembly.

**Figure 4:**
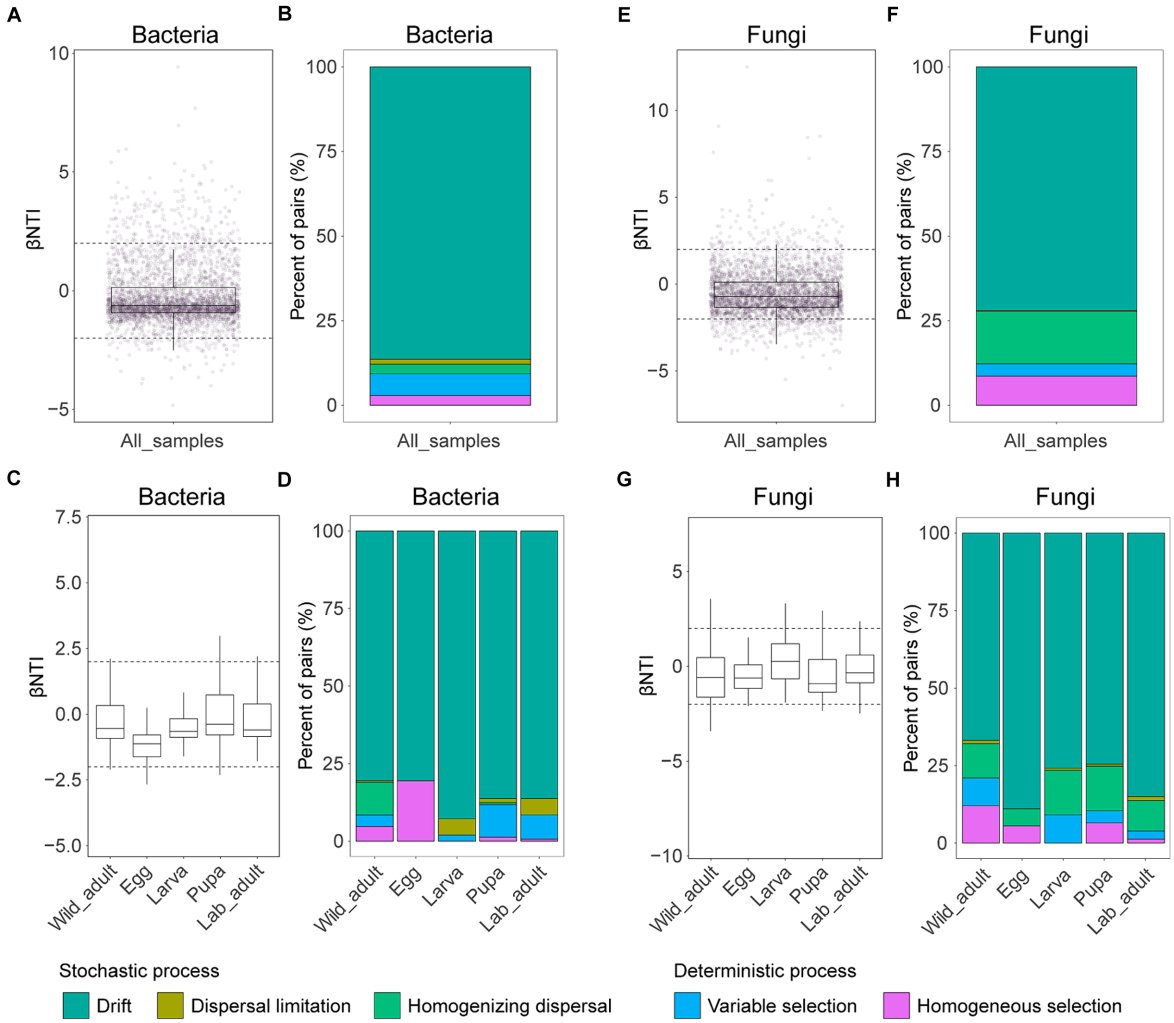
Mechanisms of bacterial **(A–D)** and fungal community **(E–H)** assembly in leaf miners. The contributions of deterministic (|βNTI| ≥ 2) and stochastic processes (|βNTI| < 2) on bacterial and fungal community assembly in all samples or different life stages. The relative contributions of ecological processes in driving bacterial or fungal assembly in all samples or different life stages.

Additionally, null model analysis supports the above findings, indicating that stochastic processes play a more significant role in governing bacterial or fungal community assembly (−2 < βNTI <2) (Figures 4A,E). Among subprocesses, drift emerged as the dominant force in shaping both bacterial (relative contribution 86.39%) and fungal community assembly (72.02%), while dispersal limitation exhibited the lowest contributions in bacterial (1.50%) and fungal community assembly (0.29%) (Figures 4B,F). Similar results were observed when each leaf stage was examined separately: drift was the primary driver in both bacterial and fungal community assembly, with relative contributions ranging from 80.53 to 92.81% and 66.84 to 88.89%, respectively (Figure 4). However, despite contributing less, the strength of other subprocesses in the formation of both bacterial and fungal communities varied among different stages (Figure 4).

### 3.4 Leaf miner-related variables and bacterial and fungal assembly

**Figure 5:**
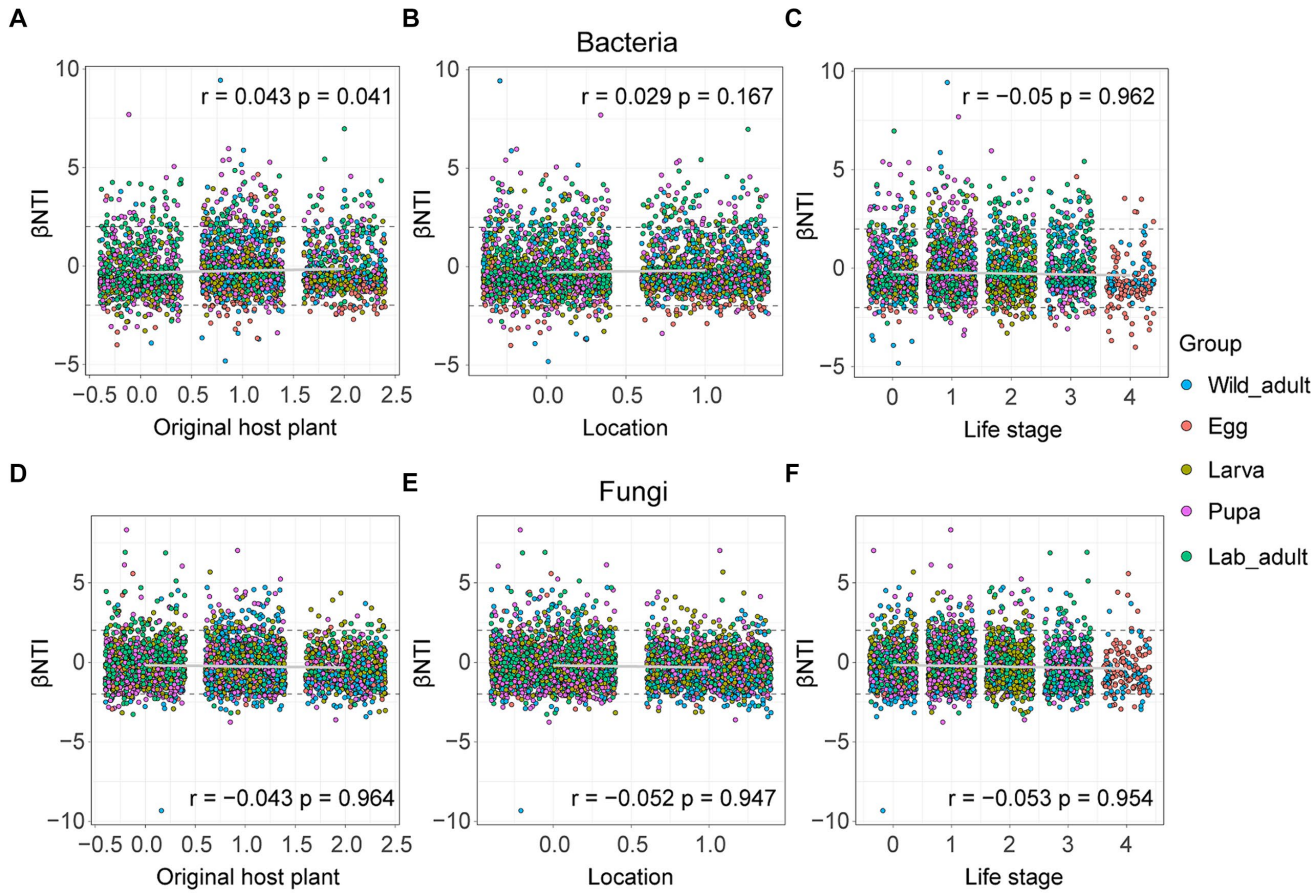
Influences of host-related variables on bacterial and fungal community assembly in leaf miners. **(A,D)** Original host plant; **(B,E)** life stage; **(C,F)** location. The *r* and *p* values were evaluated via the Mantel test.

We then examined the correction between the βNTI value of both bacterial and fungal assembly and each factor using the Mantel test, aiming to evaluate the influence of environmental factors on the microbiome or mycobiome. For bacterial community assembly, the βNTI value showed a significantly positive correlation with the original host plant (*r* = 0.043, *p* = 0.041), but showed no significant correlation with location (*r* = 0.029, *p* = 0.167) or life stage (*r* = −0.05, *p* = 0.962) (Figures 5A–C). Regarding fungal community assembly, the βNTI value exhibited weak negative correlations with the original host plant (*r* = −0.043, *p* = 0.964), location (*r* = −0.052, *p* = 0.947) or life stage (*r* = −0.053, *p* = 0.954) (Figures 5D–F). These results suggest that these factors have subtle effects on both bacterial and fungal community assembly in leaf miners.

## 4 Discussion

We demonstrate the variations in the diversity, composition and networks of both bacterial and fungal communities between wild and laboratory leaf miners. As the main drivers of community assembly, the relative contribution of stochastic processes, such as drift, was increased, causing microbiota to diverge from the wild to the laboratory.

Our findings indicate that bacterial diversity tended to decrease in leaf miners from wild to captive states, yet no distinct trend was associated with the fungal community. A similar diversity pattern of the bacterial community was observed in flies (Martinson et al., 2017; Brown et al., 2023), leaf miners (Zhu et al., 2022a), and other insects (Majumder et al., 2022; Nikolouli et al., 2022). The laboratory environment introduces dietary conditions, microbial species, and abiotic factors that alter the microbiota (Martinson et al., 2017). Among these factors, diet plays a major role, with both short-term diet shifts and long-term dietary habits affecting these communities (Huang et al., 2021; Näsvall et al., 2021; Li et al., 2024; Mugo-Kamiri et al., 2024). In our study, wild and laboratory leaf miners were reared on different plants, and the shifts in host plants possibly altered host communities. When the host undergoes novel environmental conditions in the laboratory, both host filtering and selective pressure from the environment may lead to the loss of some microbes, disrupting the balance of primitive microbial networks (Adair and Douglas, 2017). Indeed, a striking result in the current study shows that the complexity of the bacterial-fungal co-occurrence network tended to reduce. Apart from environmental variation, it is also noteworthy that the adaptation of wild insects to a laboratory environment goes hand in hand with changes in their genetic diversity and symbiont communities (Nikolouli et al., 2022). Host genetic changes may affect microbiota through host functional requirements and specific selection across co-adaptation processes (Kurilshikov et al., 2017; Ryu and Davenport, 2022). Other intrinsic factors such as host sex, physiological condition and microbial interactions may impact these microbial communities. On the other hand, our previous study suggested that leaf miners may acquire some microbes from the surrounding environment, including host plants or soil (Zhu et al., 2022a). The interaction between microbes also impacts the host microbiome (Itoh et al., 2019; Li et al., 2022a,b). We thus suggest that not only deterministic processes (e.g., host, environment), but also stochastic processes govern microbiota variation from wild to captive. Nevertheless, synthesizing the findings from previous and our current work, we argued that laboratory-reared insects harbor less diverged microbiota than wild leaf miner flies, and do not represent the full diversity of their wild counterparts.

We found that stochastic processes, such as drift, are the dominant drivers shaping microbial assembly, consistent with previous work in the honeybee and spider mites (Ge et al., 2021; Zhu et al., 2023). By contrast, our previous studies in leaf miners suggested that deterministic processes like variable selection played a primary role in driving bacterial microbial community assembly, with some influence by stochastic processes like drift (Zhu et al., 2022b). Several reasons could explain the difference in findings between the current and previous studies. One possibility is that despite the relatively weak effect, environmental variation determines the ecological process and may therefore lead to changes in the microbiome, and recent studies have provided evidence that geography differentiates the gut bacterial communities of the same honeybee species by changing the relative contribution of community assembly processes (Ge et al., 2021). Indeed, the wild leaf miner used in this study and previous research differed strongly in geography or diet. Priority effects in microbiome assembly highlight the non-negligible role of historical contingency in community assembly (Debray et al., 2022). Differences in invasive history may also occur in these leaf miners. Thus, the work suggests the importance of environment and historical contingency in governing microbial assembly, especially for the same insect species.

**Figure 6:**
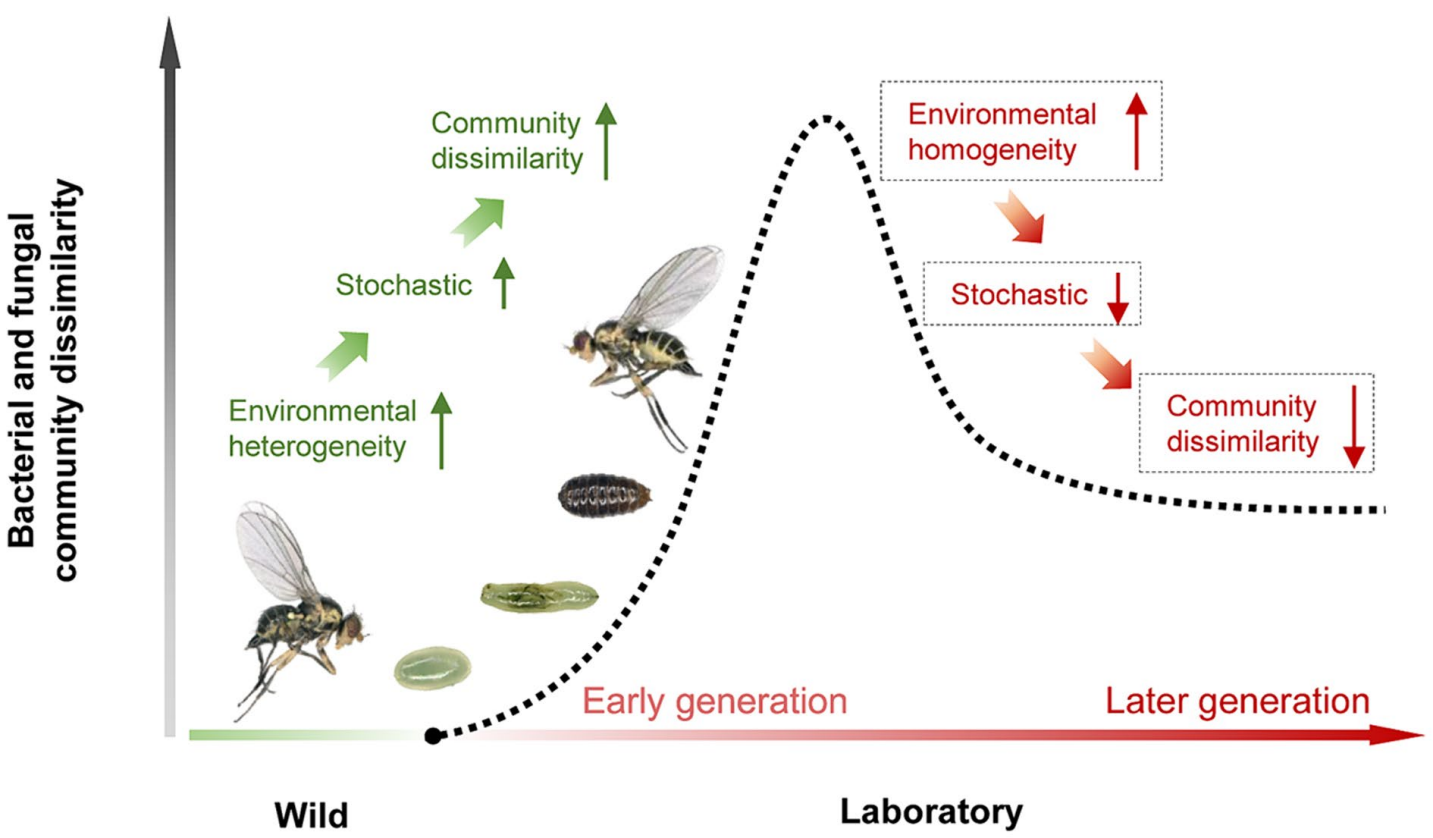
A hypothetical model of the microbiome dynamics profiles in insect short-term and long-term shits from wild to captive.

Strikingly, we compared the bacterial and fungal assembly between wild adults and the initial laboratory adults, and found that the relative contribution of drift was much higher for laboratory adults than for wild adults, while homogeneous selections were the opposite. This could explain the results of this study and other research showing a large variation of microbiota occurs in the early stage of shifting hosts from the wild to laboratory (Morrow et al., 2015; Majumder et al., 2022; Nikolouli et al., 2022; Brown et al., 2023). Our previous analyses conducted on long-term laboratory cultures provide evidence that deterministic processes mainly drive the bacterial community assembly in laboratory-adaptation leaf miners, but lack evidence of these fungal community assembly (Zhu et al., 2022a). Integrating all these empirical evidence, we propose a hypothetical model of the microbiome dynamics profiles in insect short-term and long-term shits from wild to captive (Figure 6). This model shows that (i) spatiotemporal heterogeneity drives an increase in stochastic processes linked to microbiome variance in the early stage, causing community dissimilarity, (ii) after adaptation, a stable laboratory environment leads to a decrease in stochastic processes, and an increase in deterministic processes, which may lead to microbial community similarity in the later stage, indicating that the microbiome tends to be stably maintained over time. An important next step will be to verify this model by combining neutral and null models with experimental methods.

## 5 Conclusion

In summary, our study suggests that stochastic processes, such as drift, play a principal role in determining bacterial and fungal community assembly in leaf miners during short-term shifts from the wild to laboratory conditions. Our work provides new insights into the mechanisms of microbiome and mycobiome assembly in invasive pests in the context of environmental change. Given the evident differentiation during the transfer of host from the wild, further work will explore the ecological and evolutionary consequences of this variation.

## Data availability statement

The datasets presented in this study can be found in online repositories. The names of the repository/repositories and accession number(s) can be found in the article/Supplementary material.

## Ethics statement

Ethical approval was not required for the study involving animals in accordance with the local legislation and institutional requirements because the study focuses on a pest species, therefore no ethical clearance is necessary.

## Author contributions

Y-XZ: Conceptualization, Funding acquisition, Investigation, Writing – original draft, Writing – review & editing, Resources, Validation, Visualization. X-YW: Data curation, Formal analysis, Investigation, Methodology, Software, Writing – review & editing. T-YY: Methodology, Software, Writing – review & editing. H-HZ: Resources, Writing – review & editing. T-PL: Methodology, Software, Writing – review & editing. Y-ZD: Conceptualization, Project administration, Visualization, Writing – review & editing.
