# Supplementary material for: Mechanisms of bacterial and fungal community assembly in leaf miners during transition from natural to laboratory environments
Source: Front Microbiol. 2024 Jul 18;15:1424568. doi: 10.3389/fmicb.2024.1424568 (PMC11291455; doi:10.3389/fmicb.2024.1424568)
Supplement: Supplementary file 1 [file Data_Sheet_1.docx]

**Supplementary Information for**

**Mechanisms of bacterial and fungal community assembly in leaf miners during transition from natural to laboratory environments**

Yu-Xi Zhu^a,ǂ^, Xin-Yu Wang^a,ǂ^, Tian-Yue Yang^a^, Huan-Huan Zhang^b^, Tong-Pu Li^c^, Yu-Zhou Du^a,*^

^a^ Department of Entomology, College of Plant Protection, Yangzhou University, Yangzhou 225009, China

^b^ Institute of Vegetable, Tibet Academy of Agriculture and Animal Husbandry Science, Tibet Lhasa 850000, China

^c^ Co-Innovation Center for Sustainable Forestry in Southern China, College of Forestry, Nanjing Forestry University, Nanjing, Jiangsu 210037, China

^ǂ^ These authors contributed equally to this work (Y.-X.Z and X.-Y.W.)

***Correspondence:** Yu-Zhou Du; yzdu@yzu.edu.cn

**Address:** Department of Entomology, College of Plant Protection, Yangzhou University, 88 Daxue South Road, Hanjiang District, Yangzhou City, Jiangsu Province, 225009, China

**This PDF file includes:**

Figure S1 and S2;

Tables S1 and S2.


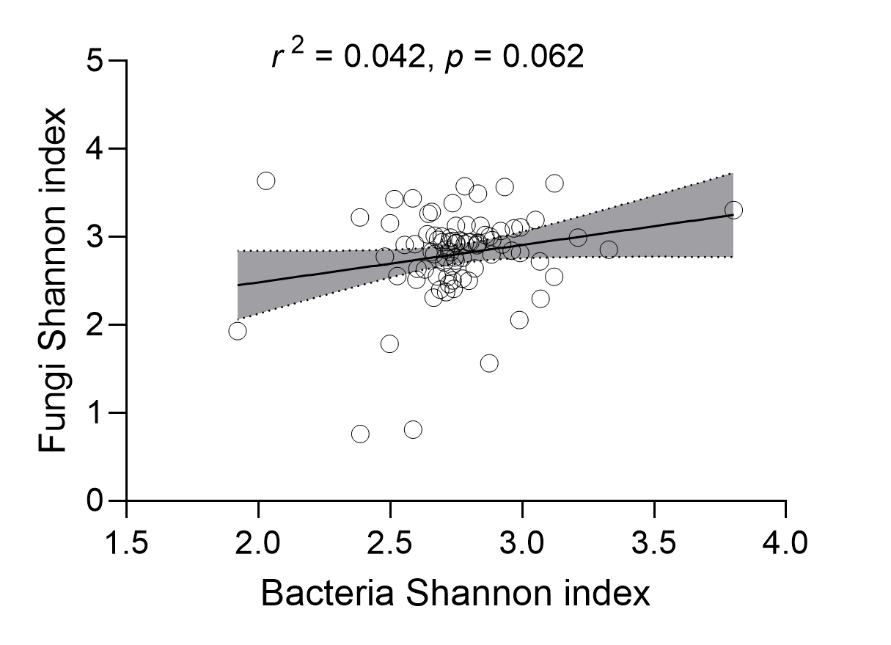


Figure S1. The relation between bacteria and fungi alpha diversity index in leaf miners.


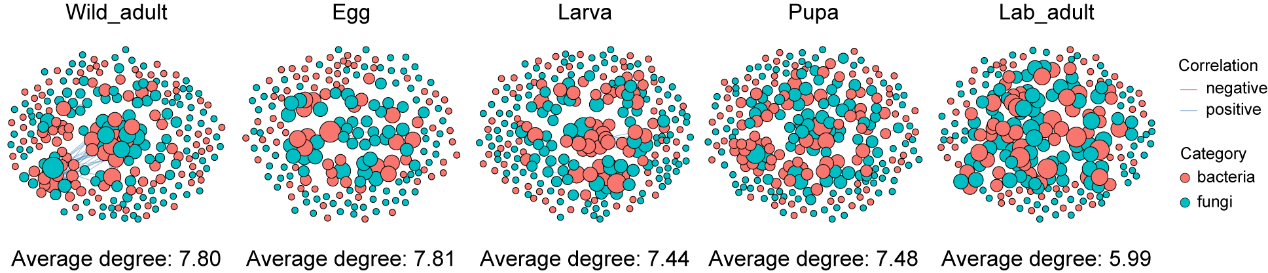


Figure S2. Co-occurrence networks of bacteria-fungi in leaf miners with each life stage.

Table S1. Bacterial or fungal co-occurrence network characteristics in each host group.

| category | property | Wild_adult | Egg | Larva | Pupa | Lab_adult |
| --- | --- | --- | --- | --- | --- | --- |
| Bacteria | num.edges(L) | 6023 | 6389 | 7085 | 10082 | 6063 |
|  | num.pos.edges | 5737 | 6388 | 6825 | 9905 | 5778 |
|  | num.neg.edges | 286 | 1 | 260 | 177 | 285 |
|  | num.vertices(n) | 476 | 373 | 481 | 487 | 488 |
|  | Connectance(edge_density) | 0.053277 | 0.09209 | 0.061374 | 0.085194 | 0.051023 |
|  | average.degree(Average K) | 25.30672 | 34.25737 | 29.45946 | 41.40452 | 24.84836 |
|  | average.path.length | 3.162881 | 1.475592 | 6.072932 | 3.033261 | 4.821535 |
|  | diameter | 8.883164 | 7.029502 | 15.38498 | 8.213335 | 11.0675 |
|  | edge.connectivity | 0 | 0 | 0 | 1 | 0 |
|  | mean.clustering.coefficient | 0.825224 | 0.918414 | 0.919796 | 0.850173 | 0.920864 |
|  | no.clusters | 2 | 20 | 17 | 1 | 13 |
|  | centralization.degree | 0.087775 | 0.136405 | 0.080293 | 0.165834 | 0.049593 |
|  | centralization.betweenness | 0.25725 | 0.011505 | 0.253242 | 0.194212 | 0.161177 |
|  | centralization.closeness | 1.552804 | 0.306347 | 1.228579 | 0.137577 | 1.308712 |
|  | RM(relative.modularity) | 4.806162 | 4.6429 | 5.748079 | 4.434711 | 5.579495 |
| Fungi | num.edges(L) | 7408 | 12865 | 7047 | 5734 | 8037 |
|  | num.pos.edges | 7368 | 12846 | 7029 | 5686 | 7985 |
|  | num.neg.edges | 40 | 19 | 18 | 48 | 52 |
|  | num.vertices(n) | 482 | 476 | 474 | 468 | 483 |
|  | Connectance(edge_density) | 0.063906 | 0.113799 | 0.062863 | 0.052472 | 0.069045 |
|  | average.degree(Average K) | 30.73859 | 54.05462 | 29.73418 | 24.50427 | 33.2795 |
|  | average.path.length | 3.077696 | 1.132191 | 5.082658 | 5.127947 | 3.536985 |
|  | diameter | 8.799932 | 8.627693 | 13.23783 | 13.64552 | 9.472918 |
|  | edge.connectivity | 0 | 0 | 0 | 0 | 1 |
|  | mean.clustering.coefficient | 0.776825 | 0.945046 | 0.817818 | 0.824247 | 0.822328 |
|  | no.clusters | 2 | 26 | 9 | 5 | 1 |
|  | centralization.degree | 0.123205 | 0.088306 | 0.072444 | 0.056736 | 0.082408 |
|  | centralization.betweenness | 0.124317 | 0.002762 | 0.141963 | 0.297024 | 0.130942 |
|  | centralization.closeness | 1.543067 | 0.193514 | 1.423704 | 1.64921 | 0.144695 |
|  | RM(relative.modularity) | 5.519876 | 8.509376 | 5.984759 | 4.910988 | 5.614001 |

Table S2. Topological properties of bacteria-fungi co-occurrence networks in each host group.

| category | Wild_adult | Egg | Larva | Pupa | Lab_adult |
| --- | --- | --- | --- | --- | --- |
| num.edges | 1615 | 1164 | 1766 | 1699 | 1201 |
| num.vertices | 414 | 298 | 475 | 454 | 401 |
| connectance | 0.018891 | 0.026303 | 0.015687 | 0.016522 | 0.014975 |
| average.degree | 7.801932 | 7.812081 | 7.435789 | 7.484581 | 5.990025 |
| average.path.length | 5.328667 | 1.5496 | 9.064067 | 6.964982 | 8.813762 |
| diameter | 19 | 4 | 24 | 20 | 23 |
| no.clusters | 17 | 36 | 17 | 15 | 15 |
| centralization.degree | 0.063434 | 0.034303 | 0.068701 | 0.071778 | 0.052525 |
| centralization.betweenness | 0.059893 | 0.002052 | 0.128563 | 0.099652 | 0.15604 |
| centralization.closeness | 1.367968 | 0.623289 | 1.473577 | 1.522176 | 1.520515 |
